# Assessing the potential for raw meat to influence human colonization with *Staphylococcus aureus*

**DOI:** 10.1038/s41598-017-11423-6

**Published:** 2017-09-07

**Authors:** Margaret Carrel, Chang Zhao, Dipendra Thapaliya, Patrick Bitterman, Ashley E. Kates, Blake M. Hanson, Tara C. Smith

**Affiliations:** 1 Department of Geographical & Sustainability Sciences, University of Iowa, Iowa City, IA 52242 USA; 2 Department of Epidemiology, University of Iowa, Iowa City, IA 52242 USA; 3 Department of Biostatistics, Environmental Health Sciences & Epidemiology, College of Public Health, Kent State University, Kent, OH USA; 4 Division of Infectious Disease, Department of Medicine, School of Medicine and Public Health, University of Wisconsin-Madison, Madison, WI USA; 5 The Jackson Laboratory for Genomic Medicine, Farmington, CT 06032 USA

## Abstract

The role of household meat handling and consumption in the transfer of *Staphylococcus aureus* (*S*. *aureus*) from livestock to consumers is not well understood. Examining the similarity of *S*. *aureus* colonizing humans and *S*. *aureus* in meat from the stores in which those individuals shop can provide insight into the role of meat in human *S*. *aureus* colonization. *S*. *aureus* isolates were collected from individuals in rural and urban communities in Iowa (n = 3347) and contemporaneously from meat products in stores where participants report purchasing meat (n = 913). The staphylococcal protein A (*spa*) gene was sequenced for all isolates to determine a *spa* type. Morisita indices and Permutational Multivariate Analysis of Variance Using Distance Matrices (PERMANOVA) were used to determine the relationship between *spa* type composition among human samples and meat samples. *spa* type composition was significantly different between households and meat sampled from their associated grocery stores. *spa* types found in meat were not significantly different regardless of the store or county in which they were sampled. *spa* types in people also exhibit high similarity regardless of residential location in urban or rural counties. Such findings suggest meat is not an important source of *S*. *aureus* colonization in shoppers.

## Introduction

The role of meat handling and consumption in the transfer of *Staphylococcus aureus* (*S*. *aureus*) from livestock to consumers is not well understood. Resistance to antibiotics such as methicillin (methicillin-resistant *S*. *aureus*, MRSA) and tetracycline has been documented in *S*. *aureus* from livestock and in individuals reporting interaction with livestock^1–9^. The primary colonization sites for *S*. *aureus* in human hosts are the nose and throat^10–13^. Hosts may become newly colonized by contact with *S*. *aureus* via other hosts or inanimate objects contaminated with *S*. *aureus*. Raw meat products have been suggested as a means by which individuals without livestock contact can potentially become colonized with *S*. *aureus* from animals, acting as vehicles of transmission of the organism from farm to the household setting. *S*. *aureus* has been found in pork, poultry, and other meats sold in commercial grocery stores and there is potential for this *S*. *aureus* to colonize humans if hygiene practices are not observed or if cross-contamination of surfaces (such as cutting boards) takes place^14–17^.

While livestock contact and meat handling are two ways by which *S*. *aureus* may colonize humans, *S*. *aureus* is also found on many other surface environments individuals come into contact with on a daily basis. *S*. *aureus* has been found on high-touch surfaces such as ATMs, mailboxes, door handles, elevator buttons, cellphones and computer keyboards^18–23^. Communities such as day-care centers, universities, prisons and gyms have been identified as sites of *S*. *aureus* colonization and transmission^20, 24–29^. Thus, the local environment of a household, other nearby households and commercial facilities may also be sources of *S*. *aureus* colonization.

Examining the genetic similarity of *S*. *aureus* colonizing humans and the *S*. *aureus* found in meat samples at the stores in which those individuals shop is one way to examine the role of contaminated meat products in human *S*. *aureus* colonization. Similarly, examining the genetic similarity of *S*. *aureus* colonizing people who live near one another answers questions about whether household location influences shared touching of *S*. *aureus*-contaminated surfaces. Sequencing of the staphylococcal protein A (*spa*) gene to determine a *spa* type is one way of approximating the genetic profile of *S*. *aureus* found in humans and meat samples. Methods developed in community ecology can determine the similarity/dissimilarity in species composition between places, both in terms of the types found and their relative proportions. Though we are examining a single species, *S*. *aureus*, the methods can be applied to examine how the composition of *spa* types found colonizing human households compares to the composition and diversity of types found in meat samples at nearby grocery stores where individuals report shopping.

Samples of *S*. *aureus* were taken from households in both rural and urban communities in Iowa and from conventional and organic/”natural” stores. We assessed the similarity of *spa* type composition from *S*. *aureus* isolated from meat samples compared to isolates colonizing humans shopping in those stores. We also determined whether residential location predicts similarity of *spa* type diversity among households. These findings inform our understanding of the role of contamination of meat products with *S*. *aureus* on the colonization of humans with *S*. *aureus*.

## Data and Methods

### Human subject enrollment and sample collection

A total of 263 participants (n = 177 adults, and n = 86 minors) as part of 95 family units from both urban (Johnson County; n = 140) and rural (Keokuk County; n = 123) counties as defined by the US Census Bureau^30^ were recruited between October 6^th^, 2011 and January 4^th^ 2012. Advertisements in a local newspaper and mailing list were used to recruit participants in Johnson County, and a previously existing cohort from the Keokuk Rural Health Study were contacted to recruit participants in Keokuk County^31^. Once enrolled, participants were instructed proper technique for self-swabbing of nose and throat^32^. On a weekly basis from January to December 2012, all adult participants provided nasal and oropharyngeal swabs, and minor participants provided nasal swabs via the use of BBL CultureSwabs with Liquid Stuart Medium (Becton, Disckinson and Company, Sparks MD, USA). Swabs were transported to the Center for Emerging Infectious Diseases lab with an icepack through the US Postal Service. In addition to weekly swabbing, participant households completed weekly questionnaires indicating where they had purchased meat products in the week prior. Questionnaire data was sent along with swabs to the lab where the data was recorded. Approval for human subject enrollment for this study was obtained from the Institutional Review Board (IRB) of the University of Iowa. All participants provided informed consent at the time of enrollment and all methods were performed in accordance with the relevant guidelines and regulations.

### Meat sample collection

Study participants were asked about the location and types of meat they purchased and consumed on a weekly basis. Based on the responses provided by the study cohort, eight stores and twelve pieces of meat from each store were sampled on weekly basis for 52 weeks from January 2012 through December 2012 resulting a total of 3290 raw retail meat samples. These stores, located in both urban (Johnson County; n = 4) and rural (Keokuk County; n = 4) were a mix of national and regional supermarkets and retail chains as well as local independent food markets and cooperative grocery stores selling antibiotic free (ABF) meat products. Sampling from a rural store in Johnson County was discontinued after eight weeks because of limited variety for sampling. Samples from three stores were from bulk, unwrapped meat while samples from the other five stores were pre-wrapped. At time of purchase, the meat samples were double bagged and transported on ice to the laboratory, where they were refrigerated until processing later on the day of purchase.

### Bacterial isolation and identification

Detailed methodology for sample collection and preparation for molecular testing are previously described^33^. Briefly, small portions (median = 117.5 grams (range = 19–388 g)) of meat samples were transferred using sterile methodology into a Whirl-Pak™ bag, mixed with 50 mL sterile peptone broth and massaged by hand (Nasco, Fort Atkinson, WI). A 50 mL portion of this wash was then combined with 50 mL of double concentration Baird Parker broth. Nasal and oropharyngeal swabs were inoculated into 5 mL of Baird-Parker broth (1X concentration with tellurite enrichment). Meat and human samples were then incubated for 24 hours at 35 °C at which time a loopful of broth was inoculated onto Baird Parker agar (BPA) plates with EY tellurite enrichment (BD) and selective MRSA agar plates (BBL CHROMagar MRSA, Becton, Dickinson and Company). These plates were further incubated for 24–48 hours at 35 °C then evaluated for bacterial growth. Presumptive S. aureus (black colonies with clear halos on BPA) and presumptive MRSA (mauve colonies on CHROMagar) were confirmed by doing the catalase test, the slide coagulase test, and the S. aureus latex agglutination assay (Pastorex Staph-plus, Bio-Rad). One colony per plate was chosen for further molecular analysis for both meat and human samples. All confirmed *S*. *aureus* isolates were subsequently stored at −80 °C.

### Molecular characterization

Genomic DNA was extracted using the Wizard Genomic DNA preparation kit (Promega, Madison, WI). The Staphylococcus protein A (*spa*) gene was amplified using SpaF (5′-GAACAA-CGTAACGGCTTCATCC-3′) and 1514 R (5′-CAGCAGTAGTGCCGTTTGCCT-3′) as described previously^34, 35^. *spa* types were assigned using Ridom StaphType software (Ridom GmbH, Germany). *spa* typing has been shown to be a robust method and comparable to pulsed field multi-locus sequence typing (MLST) in epidemiological studies^36^.

### Data preparation

#### Human vs. Meat

Given that sample sizes for households and stores varied over time, counts of samples positive for *spa* types were adjusted for multivariate analysis. Raw counts of *spa* type incidence were adjusted by sample size by store and by both store and month for human and meat samples, separately. For example, to test overall similarities of *spa* type composition between human and meat samples, adjusted-counts of types were calculated as the percentage of the number of positive isolates of a *spa* type from a store or household, given the total number of samples from the store or household. Similarly, for testing the potential for a temporal lag in the similarity of *spa* type assemblages between human and meat samples, raw counts of types were adjusted by store and by month, for both human and meat samples, respectively (equation 2). All ratios of *spa* type prevalence were multiplied by a constant (1000) in order to transform the data into integers, necessary for subsequent analysis.

1 $$\tilde{{C}_{ij}}=\frac{{P}_{ij}}{{T}_{j}}\ast c$$

2 $$\tilde{{C}_{ijk}}=\frac{{P}_{ijk}}{{T}_{jk}}\ast c$$

where: $\tilde{{C}_{ij}}$: adjusted counts of *spa* type *i* for store *j*, $\,j{\epsilon }\{1,2,3,6,7,8\}$, *P*_*ij*_: number of positive samples of *spa* type *i* collected from store *j*, *T*_*j*_: total number of samples collected from store *j*, $\tilde{{C}_{ijk}}$: adjusted count of *spa* type *i* for store *j* in month *k*, $k{\epsilon }\{{\rm{Jan}},{\rm{Feb}},\ldots ,{\rm{Dec}}\}$, *P*_*ijk*_: number of positive isolates of *spa* type *i* collected from store *j* in month *k*, *T*_*jk*_: total number of isolates collected from store *j* in month *k*, *c* = 1000, constant multiplier that converts proportional abundance to integer count.

#### Human vs. Human

Census tract was selected as the geographical unit for testing “neighborhood” effects on *spa* type assemblages among households, in that it is a spatial unit in which people often share common demographic and socio-economic characteristics. Defining neighborhood as census tract also ensured that there were enough replicates of observations so as to allow for permutation-based multivariate tests, leading to a more reliable estimate of P-value. Counts of *spa* types from households were aggregated to the census tract level and were adjusted by the total number of samples in that census tract (equation 3).

3 $$\tilde{{C}_{it}}=\frac{{P}_{it}}{{T}_{t}}\ast c$$

where: $\tilde{{C}_{it}}$: adjusted counts of *spa* type *i* for census tract *t*, $t{\epsilon }\{1,\ldots ,4\}$, *P*_*it*_: number of positive isolates of *spa* type *i* collected from census tract *t*, *T*_*t*_: total number of isolates collected from census tract *t*, c = 1000, constant multiplier that converts proportional abundance to integer count.

To examine whether the similarity/dissimilarity of *spa* type composition varied with a different definition of neighborhood, all households in Johnson County were compared to all households in Keokuk County using the same methods. Johnson County households were more urban in residential location than were those enrolled from Keokuk County, and were less likely to reside on farms or engage in livestock production. Thus, we wanted to examine whether *spa* type diversity varied across the rural/urban divide.

### Ecological distance

As a measure of compositional similarity, the original Morisita index of distance was selected based on the following desirable features^37^. Firstly, contrary to measures based on presence-absence data, such as the Jaccard and Sorensen indices, the Morisita index is abundance-based, which captures information on both species richness and species diversity, and has less bias for communities with large numbers of species or many rare species^38^. Proved by Morisita^39^ and later confirmed by Wolda^40^ and Chao *et al*.^38^, this measure is nearly independent of the number of species in a community except for rather small samples, and performs more robustly than other abundance-based measures, such as Bray-Curtis, under a wide range of sampling intensities^38–40^. Lastly, this measure is easy to interpret as it increases smoothly from zero when samples are completely distinct, to one when samples are identical with respect to species composition^39^.

The Morisita index of distance served as the basis for all the following multivariate analyses, which were used to determine whether the composition of *S*. *aureus spa* types was similar between households and meat from the stores at which they shopped, was similar between households residing in the same Census tract or similar between counties. Temporal changes of similarity in *spa* type assemblages between human and meat samples were also examined to investigate the possibility that there exists a temporal lag between meat exposure and human colonization.

### Multivariate analysis

In order to reveal the overall pattern of *spa* type assemblages between human and meat samples and among human samples, we first visually observed the distribution of *spa* types among groups in an ordination space, based on the use of non-metric multidimensional scaling (NMDS) methods^41^. NMDS is an ordination method that represents pairwise dissimilarity of objects in a low-dimensional space by assuming that the distances between sample pairs of *spa* types from different communities (stores, tracts, counties) are in rank order with their dissimilarities scaling^42, 43^. As a screening tool, NMDS can reveal possible divisions and clusters of *spa* type assemblages, and indicate grounds for further statistical tests of differences in *spa* type compositions among groups. In addition, NMDS is considered the most appropriate ordination method for community data analysis as it doesn’t assume normality of data and is proved to produce the most accurate representation of ecological data structure^44^. In this study, NMDS plots were graphed for visualizing the overall *spa* type distribution in a two dimensional ordination space by sampling group (store) and neighborhood (tract, county).

In order to quantify the effect of factors that shape group differences, Permutational Multivariate Analysis of Variance Using Distance Matrices (PERMANOVA) was used to detect differences in community composition^41^. PERMANOVA is a robust method for difference detection, using permutation procedures on multivariate analysis of variance to detect significant differences in group distances. PERMANOVA can be fitted with any actual distance measure, and therefore preserves a high degree of information of ecological distance. More importantly, PERMANOVA is suitable for any multifactorial ANOVA design, allowing for partitioning of variability for one or multiple factors and their interaction simultaneously^45–47^. The essence of PERMANOVA is to compare variability within and among groups using a pseudo *F*-ratio and *p*-value based on permutation of observations across groups^45^. Significant p-values are defined as *p* < 0.05.

The *R*-squared value is an important statistic of PERMANOVA as it indicates the effect size of factors, and is interpreted as the proportional contribution of factors to the total variance. In this study, PERMANOVA with 5000 permutations was used to estimate the proportional contribution of a single factor (store, tract, county membership) to the total variability of *spa* type assemblages. We included the total number of *spa* types in each row observation as the dependent variable, and set the independent factor as the grocery store for testing effects of retail location on *spa* type composition. Similarly, census tract and county were chosen as the independent factors to estimate effects of “neighborhood” on *spa* type compositional variability in households.

Calculation of Morisita indices, NMDS plotting and PERMANOVA calculations were all completed in the *vegan* package in R^48–50^.

### Data Availability

The datasets generated and analysed during the current study are not publicly available due to protection of human subjects information but are available as de-identified data from the corresponding author on reasonable request.

## Results

Across Johnson and Keokuk Counties, 13320 swabs were gathered from 95 households from January through December of 2012 (Table 1). Of these, 3347 (25.1%) were positive for *S*. *aureus*. From eight grocery stores, four each in Johnson and Keokuk Counties, 3290 samples were taken from meat, of which 913 (27.8%) were *S*. *aureus* positive.

**Table 1:** Sample size and *S*. *aureus* positivity for samples in Johnson and Keokuk Counties.

	Human			Meat		
	Sampling Locations (n=)	Swabs (n=)	*S*. *aureus* Positive Swabs (n = %)	Sampling Locations (n=)	Samples (n=)	*S*. *aureus* Positive Samples (n = %)
Johnson County	49	6189	1721 (27.8%)	4	1831	488 (26.6%)
Keokuk County	46	7131	1626 (22.8%)	4	1459	425 (29.1%)
Total	95	13320	3347 (25.1%)	8	3290	913 (27.8%)

In total, 126 *spa* types were observed in households during the study period. The most commonly reported *spa* types in households were t002 (n = 315; 9%) and t008 (n = 234; 7%). In total, 132 *spa* types were observed in meat samples. The most commonly reported *spa* types in meat were t002 (n = 137; 15%) and t273 (n = 87; 9.5%).

The number of samples from each store, and the associated samples from households shopping in those stores, are reported in Table 2. Observations from two grocery stores (4 and 5) were excluded from the human versus meat analysis because these stores had no households which reported shopping at that location. Finally, many households could not be linked to a store because of completeness issues in the questionnaires provided by households. All households without a linkage to a grocery store were discarded from the analysis. This left a total of 35 households to compare to meat samples.

**Table 2:** Number of *S*. *aureus* samples from stores and households who shop at those stores. Stores 4 & 5 were not included in similarity analysis.

Store	Household		Meat	
	*S*. *aureus* positive samples	Total samples	*S*. *aureus* positive samples	Total samples
1	502	1770	128	508
2	101	608	68	407
3	77	381	177	504
4	0	0	5	28
5	0	0	173	497
6	464	1376	147	534
7	288	1442	100	400
8	287	715	115	412
Total	1719	6292	913	3290

Household assignment to stores are not singular; if a household reported shopping at two stores included in the study their samples are associated with both stores. The percent of meat samples positive for *S*. *aureus* ranged from 16.7% (store 2) to 35.1% (store 3). Store 2 is an organic/”natural” foods store, while store 3 is a discount shopping chain.

Morisita indices suggest that there is high dissimilarity in human *spa* type composition when stratified by store (i.e. Morisita index closer to 0) (Table 3). Meat samples exhibited higher Morisita indices than did human samples across stores, suggesting greater overlap in the communities of *spa* types observed across stores. There were higher degrees of compositional similarity from some human/meat pairs, such as for shoppers at store 6 in Keokuk County (index = 0.36), and low degrees observed for other shopper/meat pairings (store 1 in Johnson County, index = 0.06). Interestingly, some humans had relatively high degrees of similarity to *spa* type assemblages in meat products found at stores they did not report shopping in.

**Table 3:** Morisita indices for *S*. *aureus spa* type diversity in meat samples in grocery stores and households who report shopping at those stores.

Sampling	Store	Human						Meat					
		J-01	J-02	J-03	K-06	K-07	K-08	J-01	J-02	J-03	K-06	K-07	K-08
Human	J-01	1.00											
	J-02	0.39	1.00										
	J-03	0.46	0.02	1.00									
	K-06	0.07	0.03	0.02	1.00								
	K-07	0.03	0.03	0.01	0.17	1.00							
	K-08	0.23	0.19	0.07	0.20	0.21	1.00						
Meat	J-01	0.06	0.04	0.00	0.38	0.29	0.24	1.00					
	J-02	0.25	0.14	0.07	0.45	0.07	0.33	0.72	1.00				
	J-03	0.04	0.04	0.00	0.14	0.04	0.17	0.30	0.36	1.00			
	K-06	0.14	0.19	0.00	0.36	0.15	0.27	0.82	0.80	0.35	1.00		
	K-07	0.09	0.02	0.02	0.24	0.07	0.17	0.38	0.39	0.19	0.38	1.00	
	K-08	0.10	0.03	0.00	0.23	0.13	0.14	0.55	0.48	0.43	0.59	0.30	1.00

A Morisita index of 1.0 indicates complete similarity in composition, a score of 0.0 indicates complete dissimilarity. J stands for Johnson County, K indicates Keokuk County.

**Figure 1:**
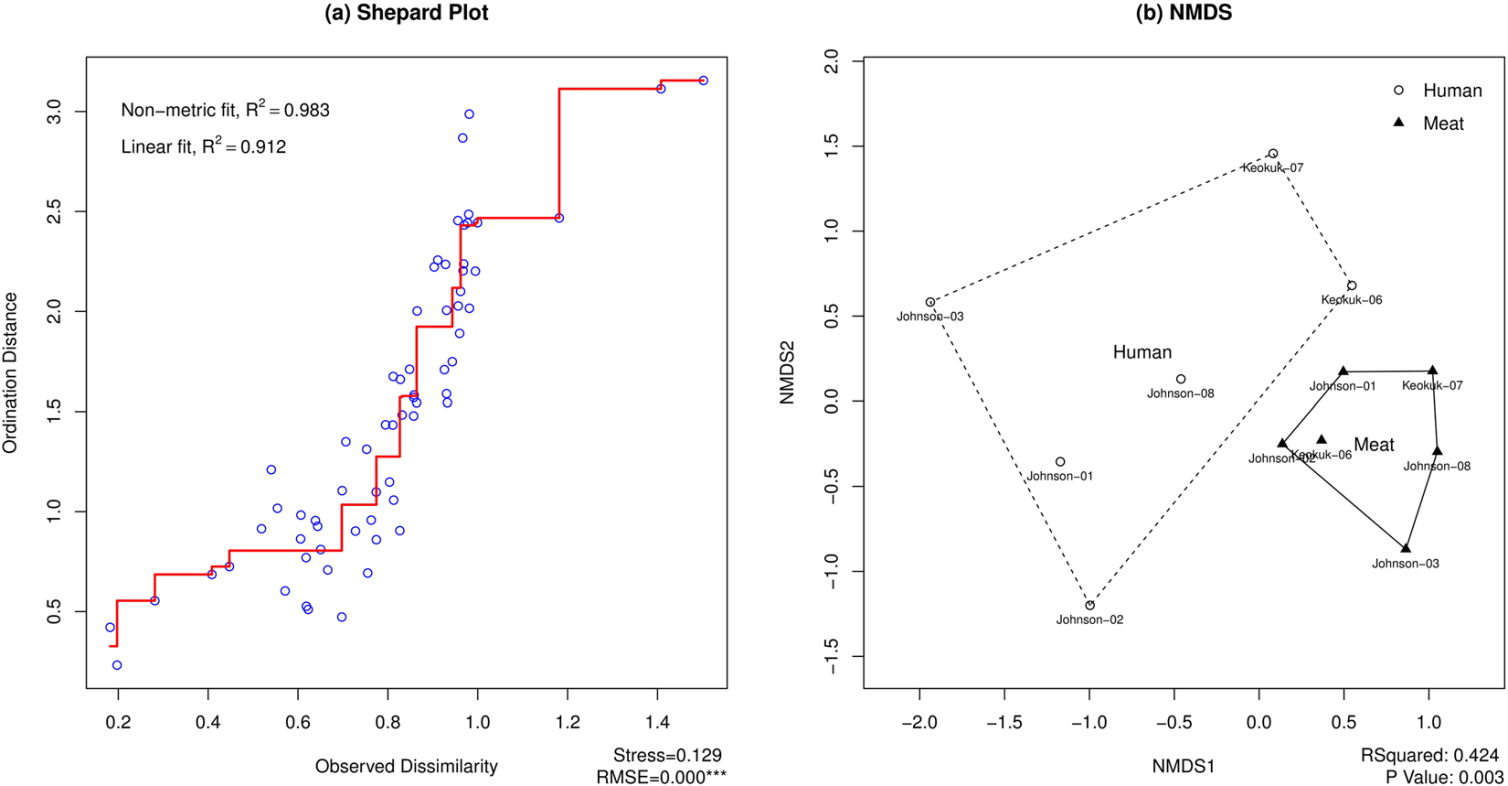
(**a**) Shepard plot visualizing ordination fit for human versus meat samples. (**b**) NMDS for visualizing patterns of *spa* type assemblages from human and meat samples. The distances between each pair of observations were plotted against their original dissimilarities. The *R*-squared and *P*-value of PERMANOVA are shown at the bottom right corner of the NMDS plot.

The Morisita indices calculated for each household and store were ordinated using NMDS, diagnostics (Fig. 1a) indicate an ordination that well-fits the observed dissimilarity: high R-squared = 0.42 and good stress that is <0.3

When visualized in two-dimensional ordination space, all household samples grouped together while all meat samples taken in stores grouped together (Fig. 1b). This grouping held even though the human and meat samples were taken from two different Iowa counties, Johnson and Keokuk. In the figure, “Johnson-08” refers to the *S*. *aureus spa* type composition of all households in Johnson County that reported shopping at store #8 (hollow circle) and composition of all the meat samples taken at that store (black triangle). The R-squared of 0.42 indicates that 42% of the variance in *spa* types is explained by the sample’s membership in either the meat or human category and that the differences observed when communities of stores and households are sorted on this factor is highly significant (p = 0.000).

**Figure 2:**
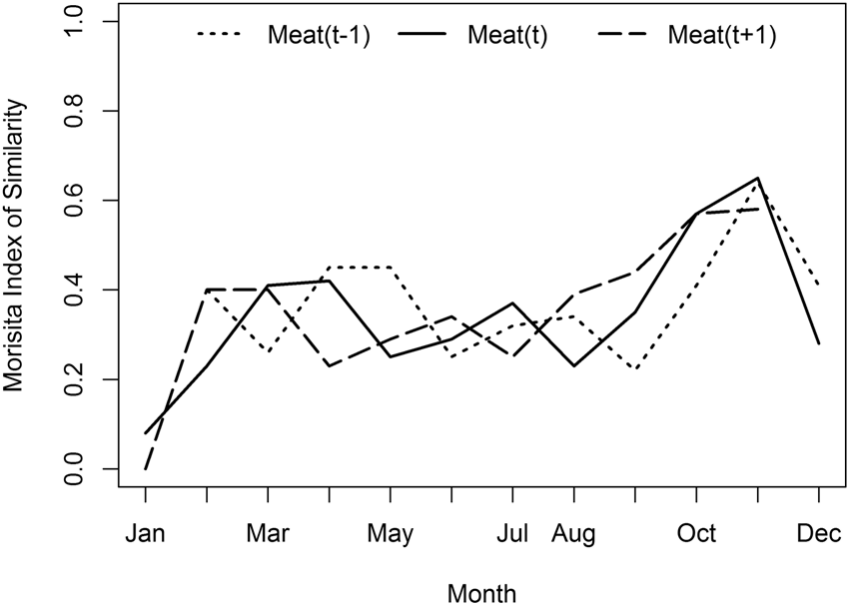
Morisita indices showing the similarity of human *spa* type assemblages to those in the meat from the stores in which they shop in the same month (t) or month prior (t − 1) or following (t + 1). Values closer to 0 indicate less similarity in composition.

To explore the potential for a lag effect, wherein the *spa* type diversity of meat samples impacts those of human populations in a subsequent time period, Morisita indices were calculated between human and meat samples at one month prior and one month following time lags (Fig. 2). Overall similarity is low, with Morisita indices of <0.4, in the earlier months of the study, rising in the final months to indices of ~0.6. There does not appear to be a temporal relationship between similarity of human samples and meat samples either from the same month or the month prior (t − 1) or following (t + 1). Instead, human samples were less similar to meat samples in the earlier months of the study and similarity in *spa* type composition in households and stores increased in the later months of the study. For detailed sample sizes by month see Table S1.

**Figure 3:**
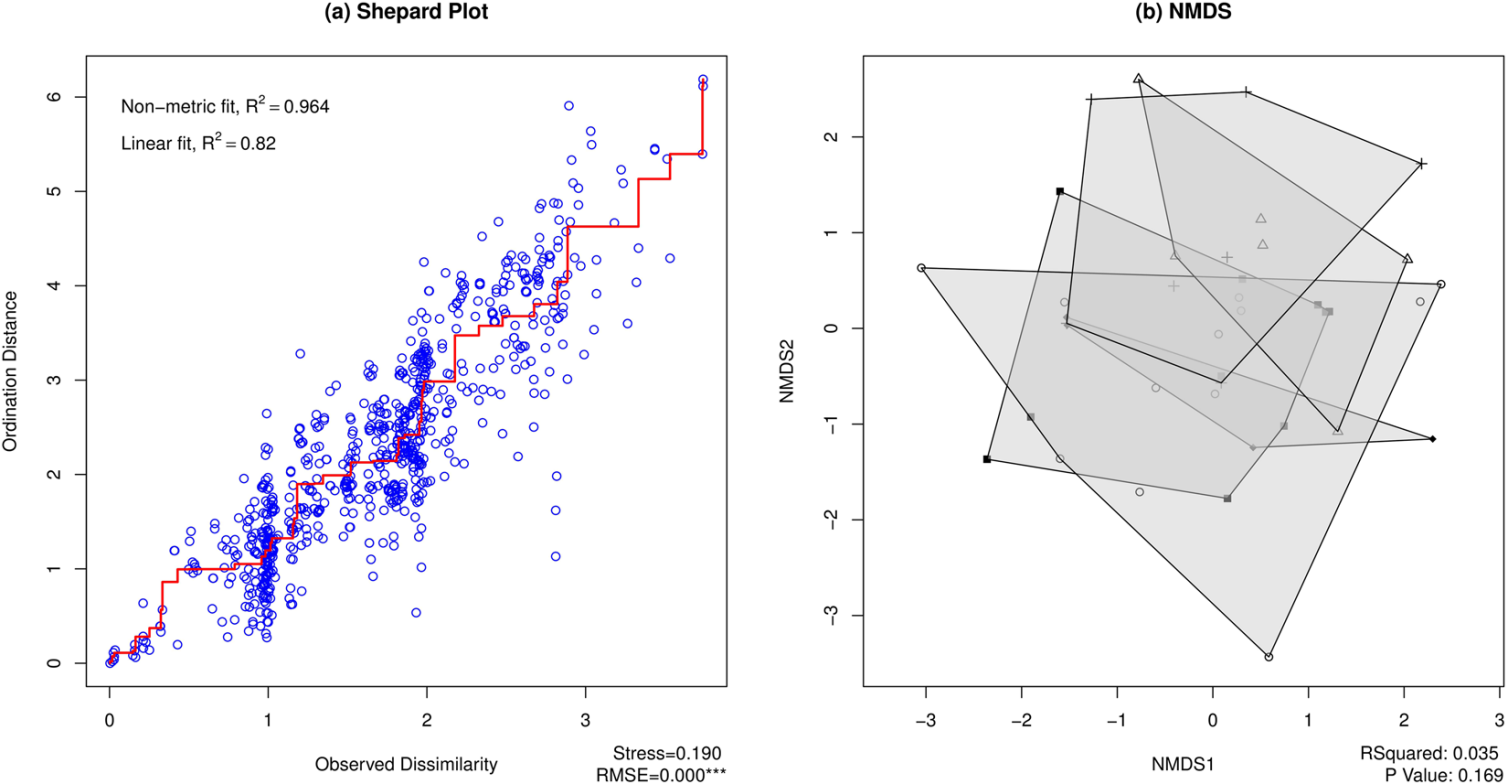
(**a**) Shepard plot visualizing ordination fit for human samples in Census tracts. (**b**) NMDS plot for visualizing patterns of *spa* type assemblages among human samples grouped by census tract. Points indicate households, shaded polygons indicate census tracts. The R-squared and P-value of PERMANOVA are shown at the bottom right corner.

To assess whether there exists similarity in *spa* types from households in the same census tracts and households in the same counties, Morisita indices were created and ordinated for households that shared these geographies. We excluded observations from census tracts in which households had zero identified *spa* types or shared no common *spa* types with others, for the reason that it was impossible to define the relationship of these disconnected sites with other sites using unconstrained ordination. Census tracts that had less than 5 households were also excluded from the analysis to ensure reliable permutation tests of within-group and across-group differences. These data restrictions resulted in a total of 40 households in 5 census tracts, 4 from Keokuk County and 1 from Johnson County, for multivariate analysis. Results indicate a high degree of similarity between *spa* type assemblages across census tracts (Fig. 3). The within-group (within tract) *spa* type composition is similar to the between-group (across tract) *spa* type composition as indicated by a low R-squared of <0.04 and an insignificant p-value (*p* = 0.17). Sorting observations (households) by community (tract) does not identify differences in *spa* type assemblage.

**Figure 4:**
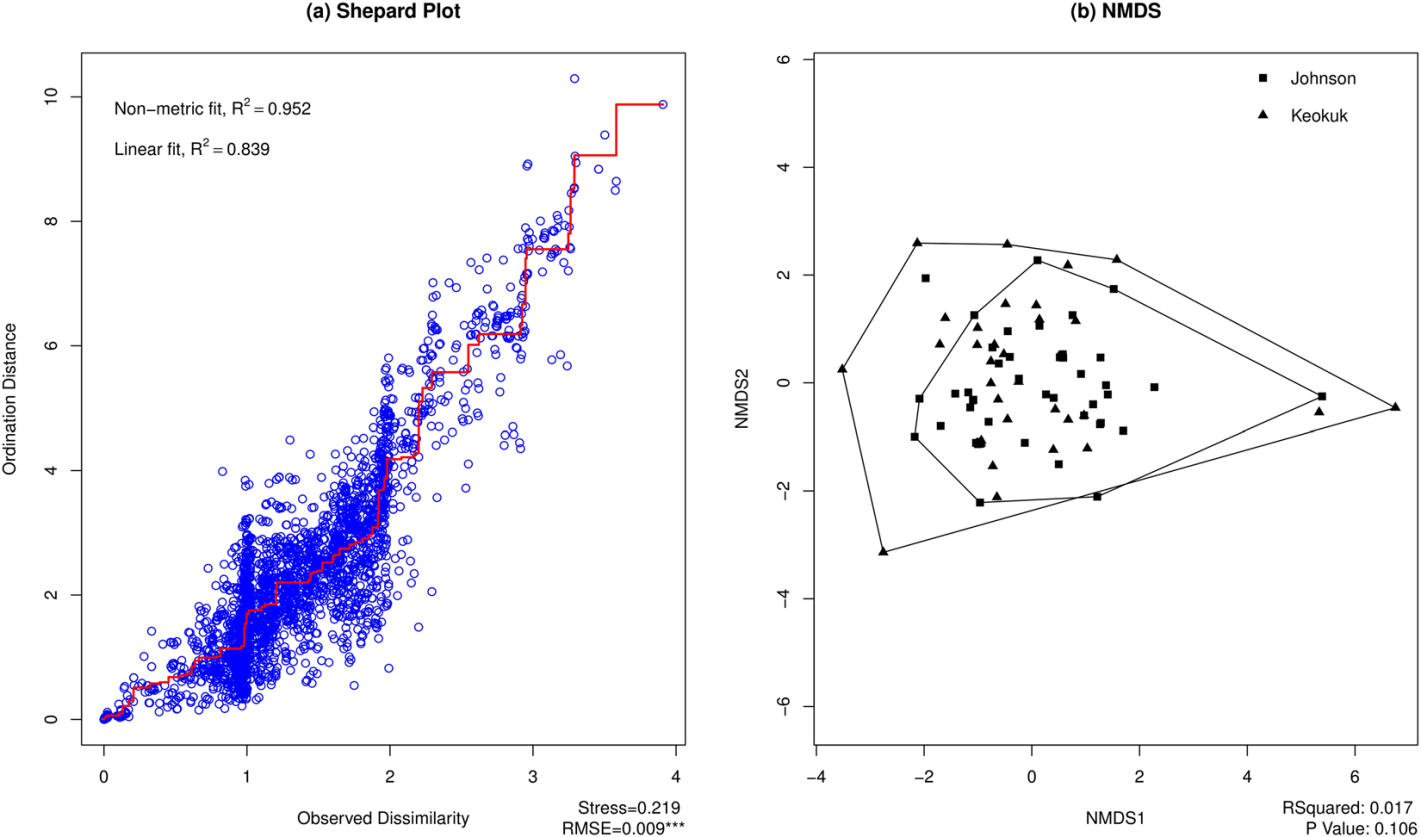
(**a**) Shepard plot of ordination fit for human samples in Johnson versus Keokuk Counties. (**b**) NMDS plot visualizing patterns of *spa type* assemblages from human samples by county. The R-squared and P-value of PERMANOVA are shown at the bottom right corner.

When the “neighborhood” definition is expanded from census tract to county, there is still a high degree of similarity in *spa* type composition across neighborhoods (Fig. 4b). After discarding samples from households that had no *S*. *aureus* positive swabs (n = 5) or that shared no *S*. *aureus spa* types with other households (n = 9) the total sample size was 81 households. The assemblages of *S*. *aureus spa* types and amounts observed in Johnson County households are not significantly different (p = 0.1) than those observed in Keokuk County households, despite very different patterns of rural/urban residence and population density and other factors. The households in Johnson County form a *spa* type community similar to that of Keokuk County.

## Discussion

The presence of *S*. *aureus* in commercially available meat products is well-established^16, 33, 51^. Particular emphasis has been placed on the detection of drug resistant variants of *S*. *aureus* and methicillin-resistant *S*. *aureus* (MRSA) in particular^14, 15, 52^. The presence of bacteria in meat products has been suggested as a possible linkage between farms and homes, providing a source of colonization and infection with *S*. *aureus* for individuals without livestock contact^53, 54^. While this has been proposed as a possible transmission mechanism, however, only limited evidence in the literature supports raw meat as a significant source of danger to human health^55–58^.

Using methods from community ecology to explore the distribution and relative abundance of *S*. *aureus spa* types in Iowa retail meat and humans, there does not appear to be a link between the types of *S*. *aureus* found in meat samples in grocery stores and human shoppers. Instead, the *spa* type composition in individual households seems to be linked to the types found in other humans in their neighborhoods, with similarity in *spa* type across census tracts and counties. This suggests there is exchange of *S*. *aureus* in ways that are more related to shared contact with surfaces outside of the grocery store environment or that there are *spa* types widely circulating and with a high degree of transmission and maintenance^59–65^. The use of all types of *S*. *aureus* for the compositional similarity analysis rather than a focus on MRSA more exclusively strengthens this finding, given that MRSA is highly clonal and less diverse than drug sensitive or other types of *S*. *aureus* ^57^. Alternately, it is possible that there are distinct communities of humans with distinct *spa* type compositions but that they are not geographically linked communities, i.e. that our assignment to tract or county does not capture the human community accurately.

Rural or urban residence does not appear to be associated with differential *S*. *aureus spa* type communities (as represented by tracts and counties, with Johnson being a more urban sample than Keokuk), nor is there differentiation by store type. These findings are similar to analyses performed on the meat and human datasets separately^32, 33^. In the first ordination the human samples grouped together to form a community while meat samples formed a separate community. Meat samples came from different types and sizes of stores. Some samples from Johnson County were taken from a store that emphasizes antibiotic free or “natural” meat production, while the remaining samples in both Johnson and Keokuk Counties came from regional or nationwide grocery chains with conventional meat supply chains. Regardless of these different sources and types of meat, the *spa* type communities in the meat samples from these stores exhibited a high degree of similarity.

Stratifying samples by month and examining for the possibility of a temporal lag in the effect of meat *spa* type diversity on household *spa* type diversity did not reveal any significant association in the *spa* types observed in meat the month before, during or following the household *spa* type diversity. Instead, overall similarity in *spa* types was low in the earlier months of the study and rose in the later months, during the winter. This suggests the possibility of seasonal shifts in the relationship between the types of *S*. *aureus* observed in meat and in humans potentially coming into contact with that meat, either as a result of seasonal behavioral change by shoppers or in livestock production. Future work can explore whether the increase in relatedness between meat *spa* type composition and human *spa* type composition in the winter is the result of decreased “competition” from environmental or community sources.

While some households in the study reported the stores at which they shop, not all households nor all stores could be included in the human versus meat/environment analysis because their shopping pattern data was not reported in the weekly questionnaires. Additionally, the relationship between households and stores is complex, as many households recorded shopping at two or more of the sampled locations. Finally, meat was not sampled from within households to establish direct linkages between *spa* types introduced in meat and those found colonizing household members. Given that the meat sampled was not necessarily the meat participants brought into the household and consumed, the sampling scheme within stores could have failed to capture *S*. *aureus* diversity that is being transferred into customer households. The wide variety of meat sampled, however, as well as the frequency of sampling, make this unlikely.

In regards to the census tract analysis, the requirement of at least five households in a tract for inclusion in the analysis meant that many household samples were lost, as that household was alone or shared the tract with only one other sampled household. Tracts are the largest unit of defined administrative boundaries below the county. Tracts could be aggregated to achieve the requisite sample size, but any such aggregation would further heighten the arbitrary nature of administrative boundaries and for the purposes of this study the clear lack of differentiation in *spa* type composition by tracts would likely hold even if more households and tracts were included.

Though this study does not definitively indicate the sources of *spa* type diversity in households, it does indicate from the data collected that it is unlikely that meat from stores is acting as the primary source. Instead, there appears to be a high degree of similarity of *spa* type diversity in the general population, across spatial scales, suggesting that shared spaces, such as schools or workplaces, are instead responsible.

## Electronic supplementary material

Table S1
