# Supplementary material for: Assessing the potential for raw meat to influence human colonization with Staphylococcus aureus
Source: Sci Rep. 2017 Sep 7;7:10848. doi: 10.1038/s41598-017-11423-6 (PMC5589955; doi:10.1038/s41598-017-11423-6)
Supplement: Supplementary file 1 — Table S1 [file 41598_2017_11423_MOESM1_ESM.pdf]

Assessing the potential for raw meat to influence human colonization with *Staphylococcus aureus*

Margaret Carrel[1,2,*]

Chang Zhao[1]

Dipendra Thapaliya[2,3]

Patrick Bitterman[1]

Ashley E. Kates[4]

Blake M. Hanson[5]

Tara C. Smith[2,3]

[1] Department of Geographical & Sustainability Sciences, University of Iowa, Iowa City, IA 52242

[2] Department of Epidemiology, University of Iowa, Iowa City, IA 52242

[3] Department of Biostatistics, Environmental Health Sciences & Epidemiology, College of Public Health, Kent State University, Kent, OH

[4] Division of Infectious Disease, Department of Medicine, School of Medicine and Public Health, University of Wisconsin-Madison, Madison, WI

[5] The Jackson Laboratory for Genomic Medicine, Farmington, CT 06032

*Corresponding author: Margaret Carrel, 305 Jessup Hall, Department of Geographical & Sustainability Sciences, University of Iowa, margaret-carrel@uiowa.edu, T: 1-319-335-0154

Supplemental materials

Table S.1 Number of *S. aureus* samples from human and meat by store and month

Store	1				2				3				6				7				8			
	Positive isolates		Total isolates		Positive isolates		Total isolates		Positive isolates		Total isolates		Positive isolates		Total isolates		Positive isolates		Total isolates		Positive isolates		Total isolates	
Sampling	Human	Meat	Human	Meat	Human	Meat	Human	Meat	Human	Meat	Human	Meat	Human	Meat	Human	Meat	Human	Meat	Human	Meat	Human	Meat	Human	Meat
Jan	2	7	4	25	0	5	0	25	0	18	0	27	11	9	24	25	0	0	4	0	0	0	0	0
Feb	62	5	164	45	16	7	56	39	5	12	31	40	26	13	91	45	19	14	94	33	20	0	49	0
Mar	100	17	292	45	26	4	102	37	16	11	61	40	46	18	136	49	37	7	161	36	39	4	87	8
Apr	70	11	225	50	16	5	92	43	7	15	41	49	49	15	148	52	31	9	136	45	30	7	68	49
May	58	10	227	40	14	3	90	32	11	9	45	40	46	10	157	43	37	4	174	36	28	8	87	42
Jun	52	8	192	44	12	4	73	32	9	12	48	40	45	6	132	44	31	11	154	36	30	14	73	44
Jul	49	9	190	52	4	2	52	39	10	13	40	50	53	13	158	52	28	11	169	45	30	13	91	55
Aug	35	9	182	36	7	6	47	27	3	13	31	40	56	7	143	38	32	6	163	31	29	8	78	41
Sep	30	12	131	38	3	4	34	28	4	17	28	40	40	13	109	40	28	7	124	30	27	21	72	41
Oct	32	15	118	50	2	7	34	38	5	22	36	49	53	15	141	53	24	9	143	39	32	17	64	48
Nov	12	14	41	40	1	3	28	30	7	16	20	39	36	13	121	40	21	10	120	31	18	11	42	36
Dec	0	11	4	43	0	18	0	37	0	19	0	50	3	15	16	53	0	12	0	38	4	12	4	48
